# Location and organization of a complex, integrated transgenic array in a *Caenorhabditis elegans* strain carrying *hlh-29p::gfp*

**DOI:** 10.17912/micropub.biology.001836

**Published:** 2026-05-08

**Authors:** Simon Anderson, Karen Barnard-Kubow, Casonya Johnson

**Affiliations:** 1 Biology, James Madison University; 2 Office of Integrative Activities, Research Capacity & Competitiveness, National Science Foundation

## Abstract

Hairy/Enhancer of Split (HES) proteins are critical for animal development and for the regulation of human diseases. The *Caenorhabditis elegans* genome encodes six HES orthologs, including HLH-29 . We used Nanopore sequencing on the MinION platform to define the location and organization of an integrated transgenic array expressing green fluorescent protein driven by the *hlh-29* promoter. The array, *ardIS501* , is at least 188.5 kb long, is inserted into Chromosome III of Bristol N2 , and contains at least 26 copies of *hlh-29 p::gfp* and 11 copies of *rol-6 ( su1006 )* . The coding sequences *Y46E12A.2 .1* and *Y46E12A.5 .1* are deleted in this strain, with no observable phenotypes.

**Figure 1. Integration and chromosomal arrangement of ardIS501:** **
A. Arrangement of *ardIS501* into Chromosome III.** This IGV screenshot shows the location of the insertion of *hlh-29 p::gfp(S65C):: unc-54* and *rol-6 ( su1006 )* into chromosome III of the wild-type *C. elegans* strain, N2 . Integration of the concatemer deleted *Y46E12A.2 .1* and *Y46E12A.5 .1* , as shown in the bottom track. Contig 37 extends 136 kb into the insertion from the upstream end and Contig 45 extends 53 kb into the insertion from the downstream end. The first 463 kb of Contig 37 and the last 332 kb of Contig 45 map to the reference genome. **B. Schematic of the Contig 37 and Contig 45 sections of the insertion.** These portions of the insertion shown comprise 188.5 kb, containing 26 copies of *hlh-29 p::gfp(S65C):: unc-54* and 11 copies of *rol-6 ( su1006 )* that are likely to be transcribed (here “likely to be transcribed” means that most of the promoter region and the majority of the protein coding region is present). An unknown amount of DNA is present between Contigs 37 and 45. The top row of features represents the forward strand of Chromosome III, the bottom row represents the reverse strand. The coordinates below the contig features refer to coordinates specific to the contig, not coordinates on Chromosome III. **C. Overlaps between assembled contigs.** (i.) Schematic of overlap between Contig 67 and 37. Contig 67 (spanning 56 kb) was called by NECAT as an independent contig. Upon manual review and interrogation with dot plots (not shown) it appears that Contig 67 is within Contig 37. (ii). Schematic of tandem overlap between Contig 64 and Contig 70. The 3'-end of Contig 64 overlaps the 5'-end of Contig 70 by 21 kb. D. **Schematic of Contig 48 and Contig 50** . Contig 48 and Contig 50 are representative of the variation among the 26 contigs that were assembled in this study. Contig 48 spans 266 kb and contains 72 copies of *hlh-29 p::gfp(S65C):: unc-54* and one copy of *rol-6 ( su1006 )* that are likely to be transcribed.&nbsp; Note that the two *rol-6* sequences near the left of Contig 48 are truncated copies of the gene that lack upstream promoter regions and are unlikely to be transcribed.&nbsp; Contig 50 spans 226.7 kb and contains 47 copies of *hlh-29 p::gfp(S65C):: unc-54* and 6 copies of *rol-6 ( su1006 )* .&nbsp; These were the two longest contigs in the assembly, at a combined length of 492.7 kb; however, neither contig could reliably be connected to the 3' end of Contig 37 or the 5' end of Contig 45, rendering us unable to confirm their location within Chromosome III.

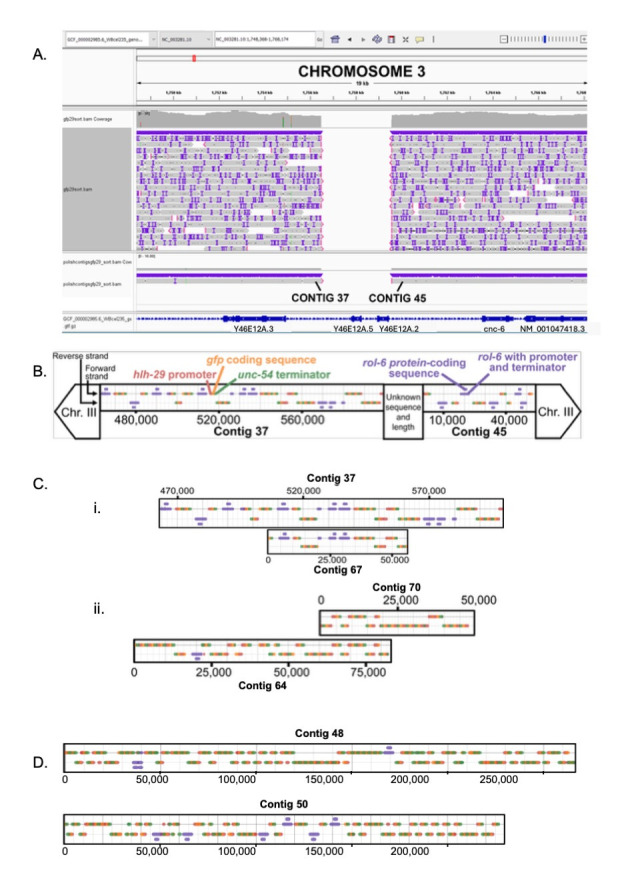



## Description

Previously, we used an uncharacterized integrated transcriptional reporter to identify cells and tissues that express the gene encoding HLH-29 (McMiller et al., 2007; Hale et al., 2014; White et al., 2012; Quach et al., 2013; Haeussler et al. 2021), a transcription factor that is responsive to Notch signaling (Neves and Priess, 2005 **)** and one of six orthologs of the mammalian Hairy/Enhancer of Split (HES) proteins. Transgenic lines were generated by microinjection of a complex array of *hlh-29 p::gfp(S65C):: unc-54* and linearized *rol-6 ( su1006 )* (Fire, 1986; Kramer et al., 1990), as previously described (Mello et al., 1991; Hobert, 2002), outcrossed six times to N2 , and then maintained as heterozygotes for greater than 100 generations. Genetic mapping and quantitative PCR to identify the site of integration and *hlh-29 p::gfp(S65C):: unc-54* copy number, respectively, were inconclusive, suggesting only that the insertion was not on chromosome I or chromosome X. In this present study, we used third generation long read sequencing on the Oxford Nanopore platform to determine the site of integration, as well as the copy number, orientation, and order of the genes contained within the array.

MinION Nanopore sequencing of genomic DNA from mixed-stage, genetically homozygous, transgenic populations produced a read length totaling 4.57 GB with 25% of the 582,770 reads exceeding 10.6 kb. The reads ranged from 1 bp to 210,269 bp, with a mean read length of 8,731 bp and a median read length of 4,864 bp. There were 200 reads over 100 kb. For reads ranging from 100 kb to 210 kb, 12 were longer than 150 kb. The complex array containing *hlh-29 p::gfp(S65C):: unc-54* and *rol-6 ( su1006 )* is inserted into Chromosome III, between bases 1,756,475 and 1,759,577 in WBcel235 ( Figure 1A ). The insertion event resulted in the deletion of 3.1 kb from the chromosome ( Figure 1A ) which deletes two genes of unknown function: *Y46E12A.2* . and *Y46E12A.5* (Hashimshony et al., 2015). Transgenic animals appear phenotypically unaffected by the deletion.

The reads assembled to 135,928 bp of the left end (Contig 37) and 52,651 bp of the right end (Contig 45) of the insertion. Contig 37 contains 20 copies of *hlh-29 p::gfp(S65C):: unc-54* and eight copies of *rol-6 ( su1006 )* that are likely to be transcribed, while Contig 45 contains six copies of *hlh-29 p::gfp(S65C):: unc-54* and three copies of *rol-6 ( su1006 )* ( Figure 1B ). Summing the regions of Contigs 37 and 45 that do not map to the reference genome gives a minimum length for the insertion of 188,579 bp, containing 26 copies of *hlh-29 p::gfp(S65C):: unc-54* and 11 copies of *rol-6 ( su1006 )* ( Figure 1B ).

In total, we assembled 26 contigs, some with significant overlap, and determined the length, *hlh-29 p::gfp(S65C):: unc-54* copy number, and *rol-6 ( su1006 )* copy number of each. We were not able to identify a single read that spanned the entire length of the insertion, nor were we able to assemble the reads and the 26 contigs to describe the full structure that links the 3'-end of Contig 37 to the 5'-end of Contig 45. However, we were able to map Contig 67 to chromosome III, within Contig 37 ( Figure 1C .i). Contig 67 spans 56 kb, containing eight copies of *hlh-29 p::gfp(S65C):: unc-54* and two copies of *rol-6 ( su1006 ).* The 26 assembled contigs ranged in length from 4.9 kb to 266 bp, containing between 1 and 72 copies of *hlh-29 p::gfp(S65C):: unc-54* , and between 0 and 8 copies of *rol-6 ( su1006 ).* We noted several other instances of overlap among the contigs; for example, there is a 20 kb tandem overlap between Contig 64 and Contig 70 ( Figure 1C .ii), and a 37.5 kb inverted overlap between Contig 57 and Contig 59 (not shown). Each of these contigs was called by NECAT as being separate sections of concatemeric chromosomal DNA, despite sharing genetic content over approximately 20kb and 37.5 kb, respectively. This high degree of similarity over a significant length of the DNA indicates that the two pairs of contigs are likely connected within a portion of concatemeric DNA, though none of the contigs could reliably be connected to Chromosome III via Contig 37 or Contig 45. It should be noted that, with the exception of Contig 67, &nbsp;the ends of Contig 37 and Contig 45 were not found to overlap to a significant degree with any of the 24 other contigs.

Finally, we noted that there was not a correlation between the number of copies of *hlh-29 p::gfp(S65C):: unc-54* and *rol-6 ( su1006 )* within a contig *.* For example, Contig 48 was the longest assembled contig, spanning 266 kb that contained 72 copies of *hlh-29 p::gfp(S65C):: unc-54* , but only 1 copy of *rol-6 ( su1006 )* . In contrast, Contig 50 spanned 226.7 kb, with 47 copies of *hlh-29 p::gfp(S65C):: unc-54* and 6 copies of *rol-6 ( su1006 )* ( Figure 1D ), failed to map to either Contig 37 or Contig 45. Collectively, based on the summation of the contigs and accounting for overlapping segments, our data suggest that the transgenic array spans greater than 454.5 kb and contains 98 copies of *hlh-29 p::gfp(S65C):: unc-54* and 12 copies of *rol-6 ( su1006 )* . However, the overlap between contigs and our inability to assemble reads to span the entire length of the insertion suggest that simple summation of the 26 generated contigs will not produce an accurate estimate of the total length of the inserted concatemer, and that computerized assembly of long reads generated via MinION Nanopore may produce separate contigs requiring human review or further processing to facilitate more complete assemblage.

## Methods

**DNA extraction**

The strain carrying an integrated concatemer of *hlh-29 p::gfp(S65C):: unc-54* and *rol-6 ( su1006 )* (McMiller et al., 2007) was created as described in McMiller and Johnson, 2005, outcrossed 10X, and maintained as a heterozygous population for greater than 100 generations (Lewis and Fleming, 1995). Prior to genomic DNA extractions, homozygous populations were generated by manual selection of hermaphrodites producing 100% roller progeny over multiple generations. DNA was extracted from approximately 200 μL packed, adult-stage hermaphrodites using the Monarch HMW DNA Extraction Kit for Tissue (New England BioLab, T3060G). DNA quality and quantity was checked using a Nanodrop spectrophotometer and a Qubit 3.0 Fluorometer.

**Library prep, sequencing, and basecalling**

The DNA library was generated using 1 μg of purified genomic DNA. The DNA was cleaned using Ampure XP magnetic beads and then prepped for MinION sequencing using the SQK-LSK110 Ligation Sequencing Kit (Nanopore). Seventy-five microliters of solution containing 12 μL prepped DNA, 37.5 μL sequencing buffer, and 25.5 μL loading beads was loaded onto a FLO-MIN106 flow cell and sequenced over 41 hours. The binary FAST5 file output from the sequencer was fed into Guppy Basecaller (Nanopore, version cpu_4.5.4_linux64) using a minimum quality score of seven.

**Mapping raw reads and assembly of de novo contigs**

Raw and polished basecalled reads (output as a FASTQ file) were mapped to the WBcel235 Bristol N2 reference genome using minimap2 (version 2.22). Basecalled reads were also assembled into de novo contigs using NECAT (version necat_20200803_Linux-amd64), with the genome size set to 100 Mb with a minimum read length of 3 kb. Read and contigs were mapped using the option “-ax map-ont”. The raw reads were deposited in NCBI's SRA ( PRJNA964757 ).

**BLAST analysis and visualization**

The original transgene was designed to contain the *gfp(S65C)* coding sequence (JX171292.1, bases 2779-3648) immediately preceded by sequences 1000 bases upstream of the chromosomal *hlh-29* coding sequence (referred to hereafter as *hlh-29* promoter: bases 17,549,640 to 17,548,640 on the X chromosome of WBcel235). The *gfp(S65C)* coding sequence is terminated by the *unc-54 (myosin-4)* terminator (JX171292.1, bases 3785-4519). The transgene was also designed to contain *rol-6 ( su1006 )* (WBVar00248869), derived from the cloning vector pRF-4 (Wormbase), kindly provided by Dr. Andrew Fire (available from Addgene, Inc., Cambridge, MA). Sequenced contigs were analyzed using BLAST (https://blast.ncbi.nlm.nih.gov/Blast.cgi) to first identify and count the occurrence of *gfp(S65C)* , and then GFP-containing contigs were analyzed to identify the locations of *hlh-29* promoters, *unc-54* 3' UTRs (terminator), and copies of *rol-6 ( su1006 ).* Raw reads, assembled contigs, and their mapping to the reference genome were visualized using the Broad Institute's Integrative Genomics Viewer (IGV). The structure of each contig (the location and orientation of *rol-6* and GFP elements) was visualized in Desmos graphing calculator using data obtained from BLAST analyses.
